# Dengue-like presentation of acute viral hepatitis in an adolescent receiving antitubercular therapy: a case report

**DOI:** 10.1097/MS9.0000000000005043

**Published:** 2026-04-29

**Authors:** Faiza Qumbar, Huzafa Islam, Ata Ul Mudassar, Muddassir Khalid

**Affiliations:** a Department of Medicine, King Edward Medical University, Lahore, Pakistan; b Department of Medicine, Nishtar Medical University, Multan, Pakistan

**Keywords:** anti-tuberculous therapy, dengue, drug-induced liver injury, tuberculosis, viral hepatitis

## Abstract

**Introduction and importance:**

Severe drug-induced liver injury, dengue, and viral hepatitis are important causes of acute liver dysfunction, but their co-occurrence in young patients is extremely uncommon and can create diagnostic challenges.

**Case presentation:**

We report a 15-year-old boy who developed severe acute hepatitis while receiving antitubercular therapy (ATT). He presented with fever, vomiting, and myalgias 4 weeks after starting ATT and was initially diagnosed clinically as dengue. Laboratory tests showed markedly elevated transaminases [aspartate aminotransferase (AST) 4172 U/l and alanine aminotransferase (ALT) 2139 U/l], hyperbilirubinemia (total bilirubin 2.03 mg/dl), and progressive thrombocytopenia. Dengue serology later returned negative, and further evaluation confirmed acute hepatitis A infection. Prompt withholding of first-line ATT and initiation of supportive care led to steady clinical improvement, with recovery achieved after 1 week.

**Clinical discussion:**

The coexistence of acute viral hepatitis and tuberculosis in young individuals is rare and may be easily overlooked, particularly when a dominant clinical or laboratory abnormality directs diagnostic reasoning toward a single etiology. Such diagnostic overshadowing can delay recognition of concurrent pathologies, especially in resource-limited or high-burden settings where certain infections are more readily presumed.

**Conclusion:**

This case underscores the importance of considering overlapping hepatotoxic triggers in young patients and highlights the need for timely, broad diagnostic evaluation.

## Introduction

Tuberculosis (TB) remains one of the most prevalent chronic infectious diseases worldwide, affecting approximately 10.8 million individuals globally in 2024, with an incidence of 134 per 100 000 population ^[^1^]^. Management requires strict adherence to a prolonged 6–9-month course of antitubercular therapy (ATT). However, drug-induced liver injury (DILI) is a significant and potentially serious complication of ATT, characterized by hepatic damage resulting from medication exposure^[^2^]^.

Concurrently, viral infections such as dengue and hepatitis A virus (HAV) continue to impose a substantial global health burden. Approximately half of the world’s population is at risk of dengue infection, with an estimated 100–400 million infections annually.

HIGHLIGHTS

- Dengue-like illness masking acute viral hepatitis in an adolescent.
- Severe transaminase elevation during antitubercular therapy.
- Thrombocytopenia contributed to the initial diagnostic anchoring bias.
- Hepatitis A confirmed after exclusion of dengue and drug-induced liver injury.
- Emphasizes broad evaluation of acute hepatitis in endemic regions.

The World Health Organization defines suspected dengue as an acute febrile illness with at least two associated clinical features, including headache, retro-orbital pain, myalgia, arthralgia, rash, leukopenia, or bleeding manifestations^[^3^]^.

HAV accounts for nearly 100 million infections worldwide each year, including around 1.5 million symptomatic cases and 15 000–30 000 deaths annually^[^4^]^.

In Pakistan, tuberculosis, dengue, and viral hepatitis are all highly prevalent, collectively contributing to significant morbidity and mortality across all age groups^[^5^]^. The overlapping clinical and laboratory features of these diseases can create diagnostic challenges, particularly when one dominant finding directs initial management^[^6,7^]^.

This case report highlights a diagnostic dilemma in an adolescent receiving ATT who presented with a dengue-like illness, ultimately found to have acute viral hepatitis, underscoring the importance of careful evaluation to distinguish drug-induced hepatotoxicity from viral infections in endemic settings.

This case report has been reported in line with the CARE checklist.

## Case presentation

A 15-year-old boy, weighing 46 kg, a known case of pulmonary tuberculosis, on standard first-line ATT for 28 days, presented to the outpatient department with a 4-day history of high-grade fever, nausea, vomiting, abdominal pain, and clinically evident jaundice.

### Investigations

Initial evaluation raised suspicion of dengue fever, and the patient fulfilled clinical criteria for dengue, with laboratory investigations showing thrombocytopenia (platelet count: 77 × 10^9^/l). Liver function tests were markedly deranged, revealing AST of 4172 IU/l, ALT of 2139 IU/l, and total bilirubin of 2.03 mg/dl.

The patient was admitted with a provisional diagnosis of dengue fever with possible DILI from ATT. However, dengue IgM serology returned negative the following day, while liver enzymes showed further worsening. Given the disproportionate elevation of transaminases, acute viral hepatitis markers were ordered, and Hepatitis A IgM was found to be positive. Relevant laboratory investigations are summarized in Table 1.

**Table 1:** Summary of all laboratory investigations of the patient.

Diagnostic investigations					
	At admission	Day 1	Day 2	Day 3	Day 11 (follow-up)
Hb (g/dl)	12	11.1	11.2	10.8	11.3
Hct (%)	34.7	33.6	34.5	32.7	35.2
WBC (×10^9^/l)	5	5.62	6.8	5.47	8.22
Platelets (×10^9^/l)	77	67	55	93	368
AST (U/l)	4172	1836	1390	919	536
ALT (U/l)	2139	1652	1210	878	452
ALP (U/l)	147	265	129	192	195
Albumin (mg/dl)	3.4	3.2	3.5	3.7	3.6
Total bilirubin (mg/l)	2.03	1.5	0.8	0.9	1
PT/INR(s)	Not done	15/1.0	16/1.0	16/1.0	15/1.0
NS1 antigen		Negative			
Dengue IgM		Negative			
HBsAg			Non-reactive		
Anti-HCV IgG			Non-reactive		
HAV IgM			Reactive		
HAV IgG			Reactive		
HEV IgM			Non-reactive		
HEV IgM			Non-reactive		

### Management

ATT was withheld immediately. Paracetamol was avoided, and the patient was managed conservatively with supportive care. He was closely monitored for warning signs, particularly in view of persistent thrombocytopenia, which reached a nadir of 55 × 10^9^/l over the next 3 days before gradually improving.

The patient showed progressive clinical and biochemical improvement and was discharged on supportive treatment, including oral and intravenous hydration adjusted to body weight and oral dextrose supplementation.

### Outcomes and follow-up

At the 11-day follow-up, the patient demonstrated complete clinical recovery with significant improvement in laboratory parameters, and ATT was safely resumed.

## Discussion

In dengue-endemic regions, patients frequently present with nonspecific febrile illnesses and overlapping clinical and laboratory features, creating significant diagnostic challenges and increasing the risk of anchoring bias toward dengue infection^[^8,9^]^.

In this case, thrombocytopenia – classically associated with dengue – acted as a misleading finding, despite being an uncommon manifestation of acute viral hepatitis. This highlights the importance of interpreting laboratory abnormalities within the broader clinical context rather than in isolation.

The coexistence of acute viral hepatitis and tuberculosis in young individuals is rare and may be easily overlooked, particularly when a dominant clinical or laboratory abnormality directs diagnostic reasoning toward a single etiology^[^10^]^. Such diagnostic overshadowing can delay recognition of concurrent pathologies, especially in resource-limited or high-burden settings where certain infections are more readily presumed.

Host factors may also play a contributory role. The patient’s low body mass index may have increased susceptibility to community-acquired infections, potentially facilitating the simultaneous occurrence of multiple infectious processes^[^11^]^. Although this association remains speculative, it underscores the importance of considering nutritional and immunological status when evaluating atypical or prolonged disease courses.

DILI remains a diagnosis of exclusion and requires comprehensive evaluation for viral, infectious, and systemic causes before attribution^[^12^]^. Premature misclassification as DILI may result in inappropriate discontinuation or continuation of medications, leading to progression of hepatic injury, delayed definitive treatment, and increased morbidity^[^13–15^]^.

## Conclusion

In dengue-endemic regions, reliance on a single laboratory abnormality such as thrombocytopenia may obscure the presence of coexisting pathologies. Careful reassessment of atypical features and early evaluation of all organ systems are crucial to avoid diagnostic delays and improve patient outcomes.

## Data Availability

Data are available on request from the authors.
